# A comparison of fit, heat stress, oxygen saturation and comfort between a novel reusable mask and disposable N95 respirator

**DOI:** 10.1371/journal.pone.0321538

**Published:** 2025-04-16

**Authors:** Maddison Strickling, Nasheed Zarif, Hoang Nguyen, Samira Moradi Khalaj, C Raina MacIntyre, Robert Packard, Guna Selvaduray, Yun Wang

**Affiliations:** 1 Department of Biomedical Engineering, San Jose State University, San Jose, California, United States of America; 2 Kirby Institute, University of New South Wales, Sydney, New South Wales, Australia; 3 Medical Device Academy Inc., Shrewsbury, Vermont, United States of America; FIOCRUZ: Fundacao Oswaldo Cruz, BRAZIL

## Abstract

The effectiveness of face masks in infection prevention depends not only on filtration technology but also on user compliance. However, existing masks suffer from limitations impacting comfort, ease of use, and communication, leading to reduced compliance, especially during prolonged use in healthcare settings. Innovations in mask design are needed to address these issues to ensure effective protection. To provide insights into novel face mask design aimed at enhancing infection prevention in healthcare settings and to introduce new evaluation methods for novel face masks, a fit and usability study was conducted with 22 volunteers, comparing a novel reusable mask, Altus Hero 1 (Hero), to N95 respirators. Subjects performed Occupational Safety and Health Administration (OSHA)-accepted quantitative fit test and usability test, and completed a post-test survey. The survey assessed communication, breathability, humidity retention, eyeglasses fitting, and long-term wear preference. Face temperature and blood oxygen levels were recorded during testing. Hero showed significantly reduced heat retention (*p*<0.05) compared to N95, aligning with survey responses indicating Hero felt cooler. No significant differences were found in blood oxygen levels between masks. Despite needing design refinements, most subjects preferred Hero for comfort and usability. This study discusses enhancements in design, fit, comfort, and materials to better meet users’ needs and ensure compliance. It highlights critical and universal design considerations for future face masks and introduces methodological innovations for evaluating mask fit and usability. The findings offer valuable insights for advancing personal protective equipment for preventing infections and future pandemics.

## Introduction

Face masks are essential for reducing exposure to infectious agents and preventing the spread of airborne diseases [1]. Users in healthcare settings, particularly healthcare workers, face unique challenges such as discomfort and reduced mask efficacy due to prolonged wear, and elevated infection risks in their high-contagion work environments [2]. While existing face masks based on mechanical barrier filtration, such as N95 filtering facepiece respirators (FFRs) and surgical masks, provide protection against airborne pathogens, they are prone to the face seal leakage due to fit and adjustability limitations [3–6]. Additionally, they often suffer from inherent limitations affecting their comfort and ease of use, particularly during prolonged wear, which may lead to reduced user compliance [7], while equal compliance for N95 and surgical masks was observed in one study [8]. Communication barriers and the visibility of mouth and facial expressions are also cited by healthcare workers as obstacles to face mask usage, with transparent masks being preferred [9]. Eyeglasses fogging is a common issue for individuals who wear eyeglasses and eye protection equipment, greatly affecting their mask-wearing experience, compliance, and performance in work and other activities. This issue particularly affects the elderly and visually impaired individuals, increasing their risk of falling due to fogged eyeglasses [10]. Therefore, innovations are needed to provide solutions that serve as effective first line of defense, thereby improving occupational and public health while helping prevent future pandemics.

Given the critical role of user compliance in ensuring the efficacy of face masks, particularly in healthcare settings where functionality and usability are equally essential, a fit and usability study of a novel face mask was conducted to explore improvements that enhance both protection and user experience. In this study, a reusable face mask, Altus Hero I (hereafter referred to as Hero), was compared to N95 respirator to assess differences in fit, comfort, heat retention, and ease of use, with the aim of identifying design enhancements that could address common limitations, including discomfort, lack of reusability, limited size options, and poor adjustability for optimal fit, and improve overall compliance and efficacy.

The Hero mask was designed to address the comfort and compliance limitations by providing a free-breathing experience. Instead of relying on mechanical barrier filtration for protection against airborne pathogens, Hero utilizes Ultraviolet-C (UVC) emitters to inactivate viral and bacterial pathogens in the unimpeded airflow both entering and exiting the mask through two disinfection capsules. This design allows the inactivation of pathogens at airflow rates of up to 80 liters per minute (L/min) (tested by Nelson Labs, CA, unpublished results from studies listed in S1 Appendix), achieving more than 3-log reductions (>99.9%) of aerosolized pathogens. For comparison, the normal breathing rate for a human adult ranges from 6 to 40 L/min, depending on activity level, from rest to moderate exercise [11]. Measured UVC exposure immediately adjacent to the skin is on the order of 5×10^−5^ µW/cm^2^ (tested by Intertek, MI, unpublished results), ensuring day-long safe wearing according to the current industry safety standard [12]. Meanwhile, since the peak wavelength of the UVC emitters is 265 nm, which is too long to ionize oxygen molecules to create ozone, the ozone generated was tested as below the 0.005 ppm detection limit of the test (tested by Intertek, MI, unpublished results) and safe to use [13]. A range of optional dust filters can be inserted into the disinfection capsules to filter out particles for applications in dustier environments. The finest filter is a mesh 1250 filter made from 316 L medical/food-grade stainless steel, which offers an effective 10 μm filtration capability. By combining the optional filters with the UVC disinfection of those ≤10 μm particles that are likely to transmit diseases [14–16], effective protection against both dusts and pathogens can be achieved as well. The Hero mask employs a transparent facepiece to facilitate communication and is made reusable through simple disinfection procedures, such as using disinfectant wipes.

To assess the Hero design, N95 respirator was employed as the comparison standard, as it is one of the most widely used FFRs in healthcare settings. Given that Hero was designed to address the healthcare needs, comparing its performance to the N95 allows for a meaningful evaluation of improvements in comfort, usability, and protection. Since the efficacy and safety of the Hero mask have already been tested and demonstrated (as summarized in the preceding paragraph), this study focuses specifically on its fit and usability in comparison with N95, with implications for future mask design improvements.

Usability studies can provide valuable feedback for the design process as they help validate designs based on user experiences [17]. Mask properties such as user comfort, breathability, temperature, and fit are the primary indicators for user approval and compliance [5]. While addressing the need to develop novel face masks for higher efficacy, evaluation methods for traditional face masks are sometimes insufficient or inapplicable. Herein, we modified evaluation methods for fabric-based face masks to evaluate novel mask designs that incorporate new technologies such as the Hero mask. A quantitative fit test was conducted using a modified Occupational Safety and Health Administration (OSHA) N95 respirator quantitative fit test protocol [18] to evaluate the sealing effectiveness of the Hero mask. A comparative usability test between Hero and N95 was performed followed by a questionnaire-based survey. The survey gathered participants’ personal assessments of donning and doffing, ease of use, and comfort during the various activities for both Hero and N95. Through this comparative study, we aim to develop new approaches for evaluating novel face mask designs and provide design considerations that can be universally applied to enhance user experience and compliance in future face mask development.

## Materials and methods

### Hero masks and N95

**Fig 1:**
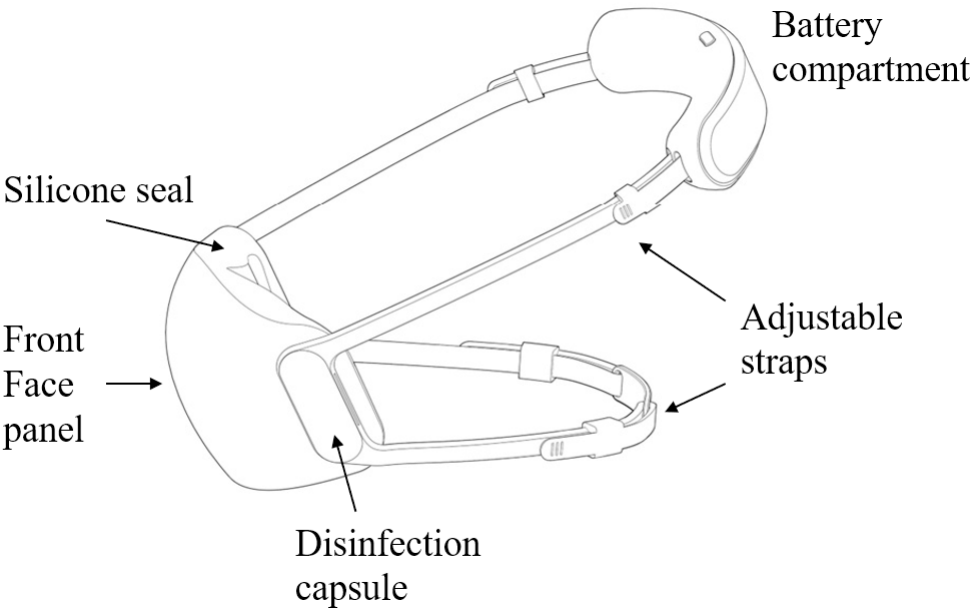
Major components of the Altus Hero I (Hero) mask. The Hero mask is a battery-powered reusable mask. It consists of a transparent front face panel surrounded by a silicone seal to prevent the leakage of non-disinfected air. It utilizes solid-state Ultraviolet-C emitters to inactivate viral and bacterial pathogens in the unimpeded airflow both entering and exiting the mask through disinfection capsules on the two sides of the front panel. There are Velcro adjustable straps over the head and ears to fasten the mask to the face. There is a light-emitting diode indicator located on the battery compartment that will indicate the system mode and battery status with different colors.

The Hero mask is a reusable mask that covers the nose and mouth to protect the wearer against bioaerosol particle transmission. A schematic showing the major components of the Hero mask is presented in Fig 1. It consists of a transparent front face panel surrounded by a silicone seal to prevent the leakage of non-disinfected air. There are Velcro adjustable straps over the head (head-strap) and over the ears, which are connected at the back of the head to secure the mask on the head. The straps are connected to the front panel through loop attachments bonded to the front panel. Two disinfection capsules with air inlets and outlets are also attached to the front panel. The Hero mask relies on solid-state UVC light emitters with 265–275 nm peak emissions to inactivate airborne pathogens at typical breathing rates. Electric power is supplied through a battery compartment located at the back of the head when wearing. When the battery is inserted into the battery compartment, the mask will be automatically turned on. The inhaled and exhaled air will go through the disinfection capsules through the inlets and outlets.

There is a light-emitting diode (LED) indicator located on the battery compartment that will indicate the system mode and battery status with different colors, including inhale protection (air influx disinfected, blue), bi-directional protection (air influx and efflux disinfected, blue and green, respectively), or warning (amber) which is coupled with voice and haptic warnings to indicate low battery level or component malfunctions.

Four Hero mask sizes (XL, L, M, S) with matching adjustable straps and one model of universal 3M particulate respirator N95 (model #:8210) were tested. The Hero mask’s various sizes were designed based on the measurements from standard NIOSH face-type classification [19]. A set of Hero masks of different sizes were modified in order to conduct the OSHA fit test. Each modified Hero mask has a sampling probe connector, installed on the mask, that allows the test probe to sample the air from the inside of the mask. Probe attachments (TSI sampling adapters) from TSI Inc. (Shoreview, MN) that permit fit testing in different respirators were attached to the probe connector during the test. Each modified Hero mask also has two connectors to air purifying respirator filters, as the modified units were not battery-powered to avoid interferences to the fit test due to the airflow. In addition, as Hero utilizes UVC emitters to inactivate pathogens in the unimpeded airflow both entering and exiting the mask through two disinfection capsules, the modified Hero mask allowed us to decide whether the Hero mask provides a seal, in a level comparable to the N95, to ensure that air flows only through the designated disinfection capsules and not from unintended locations. Air purifying respirator filters from 3M (St Paul, MN), 3M™ 7093 Particulate Filter, P100, were attached to the connectors during the fit test.

### Subjects

We recruited 22 volunteers, including students and staff members, from San Jose State University. Subject inclusion/exclusion criteria were determined to be 18 years of age or older with no facial hair. Given the objective of this study focuses on uncovering usability issues for future design improvements, the sample size was decided following the established practices in usability engineering, as detailed in the FDA guidance, ‘Applying Human Factors and Usability Engineering to Medical Devices’[17] and industry standards such as ISO 62366–1. The guidance and industry standards support the use of relatively small samples (in the range of 15–20 subjects) for formative usability evaluations, where the objective is to identify and address potential use errors and design issues. As the first stage of the test, volunteers were additionally screened for a history of significant respiratory conditions which may interfere with the results of the study. Ideal volunteer demographics would have an even spread of users who did or didn’t wear eyeglasses, gender, age, and face size. This would allow for a range of user factors to be evaluated during the study. The recruitment period for this study was from February 22^nd^, 2022 to April 14^th^, 2022. A preliminary screening questionnaire was given to interested individuals who replied to the volunteer recruitment, and the test subjects were selected based on the inclusion/exclusion criteria.

### Study structure

The study was designed to be conducted over two days for 16 hours total. Each day was broken into five 1.5-hour test sessions. Each session could accommodate up to three subjects simultaneously based on subject availability. As each subject arrived at the study location, they would be asked to read over and sign the informed voluntary consent document (written consent document). After they consented to participate, the subjects would be directed to one of the three tests, Hero fit test, N95 usability test, or Hero usability test, according to the availability of each test upon the subject arrival. After each test, the subjects would be instructed to proceed to the next test until they had completed all three. The order of wearing either Hero or the N95 was randomly assigned in this case for each subject.

### Hero fit test

The quantitative fit test was conducted by Safety Compliance Management, Inc. (San Ramon, CA), following the Occupational Safety and Health Administration (OSHA) Fit Testing Procedures (1998). The detailed test protocol is presented in the S2 Appendix. The equipment used was a TSI Portacount Pro 8038 Fit Tester (TSI Incorporated, MN). The built-in N95 Companion^TM^ was used to focus on particles that entered via leakage rather than those penetrating through the filter, evaluating Hero’s fit and seal. For the fit test, an overall fit factor (the mean of fit factors obtained from all test exercises performed by a subject, see S2 Appendix) of 0–200+ was given for each subject while wearing Hero. The overall fit factors over 200 were displayed as 200+ on the TSI Portacount Fit Tester. A number equal to or larger than 50 was defined as a “PASS” criterion for the fit test. Conversely, an overall fit factor less than 50 was defined as a “FAIL” criterion for the fit test, following the instructions of the Fit Tester.

### Hero and N95 usability tests

The Hero masks tested in the usability test were battery-powered finished products. The usability comparison test was conducted by comparing the Hero masks with 3M particulate respirator N95 (model #: 8210). The front panel and silicone seals of each Hero mask were wiped down three times with fresh disinfectant wipes (Clorox brand, Bleach Free Disinfecting Wipes) between subjects. Each subject used a fresh wipe which was discarded after use. Each subject used a new disposable N95 respirator which was discarded after use. The usability tests were conducted in the same room with a climate-controlled environment.

The procedure of the Hero usability test was: 1) The subjects were provided with background information and video demonstrations of the don/doff of Hero. An in-person demonstration by the test moderator was also performed immediately prior to the actual test. 2) Each subject donned a Hero mask to perform a self-fit check to ensure that the silicone seals around the face were tight and there was no air leak. After the self-fit check, a proper Hero mask size for each subject was decided and the mask was properly worn. The baseline blood oximeter reading using MightySat™ Rx Fingertip Pulse Oximeter (Masimo Corp., CA) and face temperature reading through thermal imaging using Seek Thermal Compact thermal imaging camera (Seek Thermal Inc., CA) for each subject was recorded. During the face temperature measurement, the subjects were instructed to breathe normally. A test moderator captured thermal images 1.5 meters away from each subject using the thermal imaging camera. While the camera was showing live thermal images, the highest temperature over the mask-wearing region observed was recorded for analysis. Face temperature measurement was conducted in the same way throughout the test. 3) The subjects were asked to wear the mask while performing a variety of short tasks that included sitting (10 minutes), walking (one minute), talking (during the sitting and walking), and performing an exercise (jumping jacks for one minute) to elevate respiratory rate and potentially heat accumulation on the mask-wearing region [20]. Immediately after the exercise (jumping jacks) and two minutes after the exercise, the blood oximeter readings and the face temperature readings were recorded. 4) LED indicator warnings, if any, were recorded by a test moderator while the mask was being worn by the subjects.

The procedure of the N95 usability test was: 1) The subjects were provided with N95 wearing instructions [21] which cover the Cal/OSHA (8 CCR Section 5144) training requirements for disposable N95 filtering facepiece respirator users. 2) Each subject donned a N95 (3M, model #: 8210). The baseline blood oximeter readings and face temperature readings were recorded using the MightySat™ Rx Fingertip Pulse Oximeter (Masimo Corp., CA) and Seek Thermal Compact thermal imaging camera (Seek Thermal Inc., CA), respectively, same as the Hero test. 3) The subjects were asked to wear the N95 while performing a variety of short tasks that included sitting, walking, talking, and exercising, the same as the Hero test. Immediately after the exercise and two minutes after the exercise, the blood oximeter readings and the face temperature readings were recorded.

We also studied the eyeglasses fogging comparison between Hero and N95. Both the seal and comfort are affected by how well the mask fits for different shapes and sizes of the subjects’ faces. The subjects who wore eyeglasses were asked to wear their own eyeglasses during the test.

### Post-test questionnaire

After the completion of all three tests, the subjects would be asked to fill out a questionnaire which is presented in S3 Appendix. The survey was self-administered. In the questionnaire, we asked the subjects about their preferences for Hero or N95 in several categories that were identified as indicators of comfort and functionality relevant to the comparison of the two, including breathability, heat retention, moisture retention, eyeglasses fogging, communication, and long-term comfort [5]. We evaluated the subjects’ preferences with a 5-point scoring system used in six questions. A score closer to 1 meant a greater preference for Hero, closer to 3 meant less preference for either mask, 3 meant no preference, and closer to 5 meant a greater preference for N95. The accumulation of humidity inside the mask can lead to a damp or stuffy feeling. The moisture retention was assessed through subjective user feedback on perceived humidity. Subjects were asked to report their perception of humidity inside the mask, whether they felt excessive moisture buildup over time, and whether the mask interior felt persistently damp. The heat retention was assessed based on both the temperature reading using the thermal imaging camera and the subjective user feedback on perceived warmth. Subjects were asked to report their perception of warmth inside the mask, whether they felt heat buildup over time, and how it affected their comfort. In addition, the subjects were asked whether they were able to hear and recognize different types of the voice and haptic warnings while wearing Hero.

### Data and image analysis

We conducted Analysis of Variance (ANOVA) tests and two-tailed paired *t*-tests to analyze the temperature and blood oxygen saturation data. We used ImageJ to analyze the thermal images obtained during the face temperature measurement. During the tests, we observed that the shape of the face affected the fit and comfort of using Hero. Due to time constraints and subject availability, instead of anthropometric measurements, the test moderators recorded the observation of face shapes during the test. To address the limitation and better characterize the face shapes for guiding the design of future masks, we measured the total facial height and bizygomatic width based on the thermal images using ImageJ and calculated the facial ratio (height/width ratio). A normal face would have a facial ratio between 1.6–1.699, a short face would have a ratio smaller than 1.6 ratio, and a long face would have a ratio over 1.699 [22]. Gender was also evaluated as a potential factor for varying the face temperatures and user experience. Additionally, we used ImageJ to analyze the mask-wearing region to study heat intensity and distribution. Using the color histogram feature, we extracted the red channel from the images, which indicates the high temperature. We obtained the mean intensity values of red color and standard deviation (SD) of the intensity using ImageJ built-in functions, indicating the heat intensity to characterize heat retention. Through the analysis, we were able to provide a more comprehensive look at the temperature each subject was experiencing while wearing the different mask/respirator.

### Ethical approval

This study is approved by San Jose State University (SJSU) Human Subjects Institutional Review Board (IRB). Tracking Number: 21245. Date of Approval: 12/15/2021. The written informed consent to participate in the study was acquired from each participant. The use and dissemination of study results that are not associated with participants’ identities were approved by SJSU IRB. The results of the study are not associated with the participants in any way, and the results do not include any personal data of the participants. No records are included here that allow participants’ names and identities to be associated with their responses in the study or on the survey. Participants’ responses in the study and on the survey were anonymous.

## Results

### Subject demographics

The age of the subjects, presented in S1 Fig, ranged from 18 to 47, with a mean of 23 and a median of 20. The subjects include students and staff members from San Jose State University. Of the 22 subjects, 64% (14/22) are female, and 36% (8/22) are male. Five subjects, 23% (5/22) of the subjects, wore their own eyeglasses to the study.

### OSHA fit test for Hero

Among the 22 subjects, there was one failed fit test (Subject #18) for Hero, based on the defined pass criterion (see ‘Hero fit test’ section), due to the reason that the smallest available size (S) might be slightly too large for the subject. It was reported that although the mask was securely fastened and stayed in place, there was a small gap felt between the chin and the bottom of the mask. When moving the head up and down, and bending over during the fit test, air was felt to be escaping from the mask. For the rest 21 subjects, with a proper size of the mask, the overall fit factors (see S1 Table) obtained indicated the pass of the test. Hero showed a good fit test pass rate (21/22) to be comparable with N95 [23]. In addition, the test results demonstrated that Hero provided a seal at a level comparable to the N95, ensuring that air only flows through the designated disinfection capsules and not from unintended locations.

### Usability comparison

**Fig 2:**
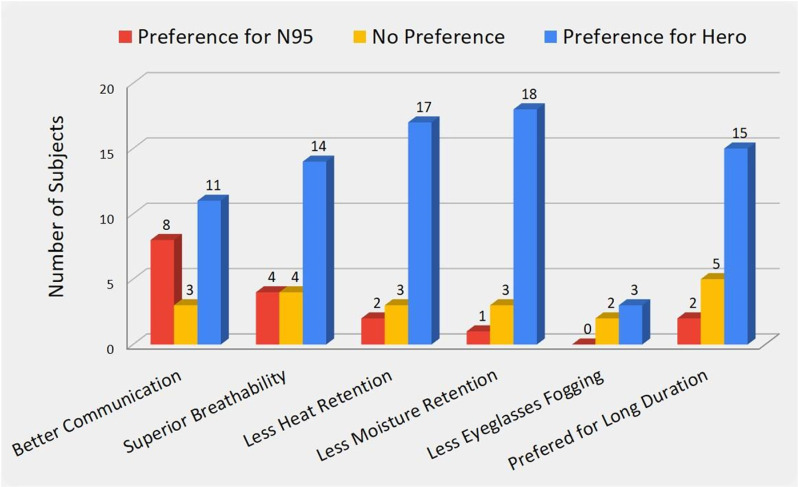
Mask preferences of subjects for a variety of indicators of comfort and functionality.

The responses from the subjects are shown in Fig 2.The ratings given by the subjects indicated an overall greater preference for Hero. Communication (talking and listening while wearing Hero or N95) comparison provided the greatest difference in preferences. For communication, 50% of subjects preferred Hero while 36% preferred N95, and 14% had no preference. Hero was preferred by 63% of subjects, based on the ease of breathability. This can be largely attributed to the design of Hero. There is a large space between the transparent front panel and subject’s mouth and nose, and there is free airflow entering/exiting the space, not retaining the moisture from breathing which is common when using an N95. Additionally, 77% of the subjects thought Hero had less heat retention and 82% felt Hero had less moisture retention in the mask, meaning that they felt the inside of the mask remained cooler and drier during use. There were 22% of subjects wearing eyeglasses during the study, among which 60% reported less fogging with Hero. Some subjects had difficulties fitting their eyeglasses over Hero due to the positioning of the mask itself. The mask sat higher up on users with short faces and did not provide enough room for their eyeglasses to fit over the mask, particularly the eyeglasses with larger rounder frames. As a final metric for usability, subjects were asked which mask was preferred for a long duration wear. From this parameter, 68% of subjects preferred Hero, 9% preferred N95, and 23% had no preference.

Table 1 summarizes the average scores from all, female, and male subjects, respectively. The average scores were based on the 5-point scoring system mentioned above. The scores for “less heat retention” and “preferred for long duration” are significantly different between the responses from female and male subjects (*p*<0.05), while the scores for other parameters are not. Female subjects had an average score closer to 1 (greater preference for Hero) than male subjects for all parameters, indicating a greater preference for Hero in this group.

**Table 1:** Average scores indicating the preference between Hero and N95 from all, female, and male subjects, respectively.

Subject group	Better communication	Superior breathability	Less heat retention	Less eyeglasses fogging	Less moisture accumulation	Preferred for long duration
All	2.7 ± 1.4^a^	2.3 ± 1.2	2.0 ± 1.1	2.0 ± 1.0	1.6 ± 0.9	2.1 ± 1.2
Female	2.6 ± 1.6	2.0 ± 1.2	1.6 ± 0.8	1.8 ± 1.0	1.4 ± 0.9	1.8 ± 1.2
Male	2.9 ± 1.1	2.8 ± 1.0	2.5 ± 1.3	3.0^b^	1.9 ± 0.8	2.8 ± 1.0

^a^A score closer to 1 meant a greater preference for Hero, closer to 3 meant less preference for either mask, 3 meant no preference, and closer to 5 meant a greater preference for N95. Same for all scores presented in the table.

^b^Only one male subject wore eyeglasses during the study.

### Facial ratio of the subjects

**Fig 3:**
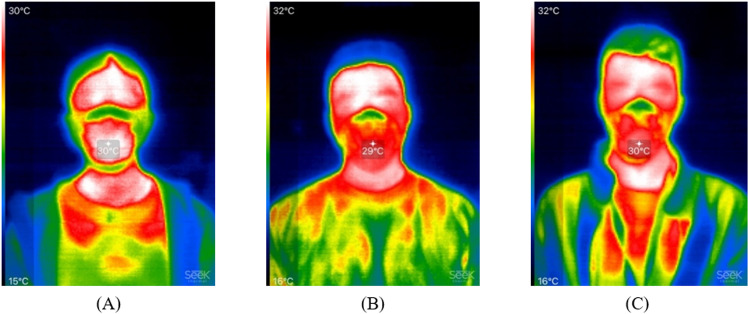
Examples of thermal images used for facial ratio measurements. Thermal images of: (A) Subject #7 with a facial ratio of 1.30 which was characterized as short face (ratio < 1.6), (B) Subject #4 with a facial ratio of 1.64 which was characterized as normal face (ratio range 1.6 to 1.699), and (C) Subject #6 with a facial ratio of 1.95 which was characterized as long face (ratio > 1.699). The facial ratio was calculated as vertical direction measurement (total facial height) obtained in pixels over horizontal direction measurement (bizygomatic width).

S1 Table shows our measurement results taken from the thermal images, with three examples presented in Fig 3. The mask size for each subject is presented in S1 Table as well. There were two subjects (#5 and #14) who were better fitted into a mask (e.g., sized L for #5) with adjustable straps paired with a different-sized mask (e.g., L-sized mask with straps originally paired with M-sized masks for Subject #5). It is noted that since the measurement was based on the thermal images, the pixel number does not represent the actual dimension of the faces. It is also noted that due to the limitation of using thermal images not actual photos of the subjects, the measurement was affected by the quality of the thermal images. Though the test moderators took the thermal images at the same distance away from the subjects, fine movements and head orientation differences might affect the facial height and width shown in the images. In addition, since the mouth and chin region were covered by the masks, the total facial height was obtained between the hairline (the top border of the red region at the forehead) and the bottom border of the red mask-wearing region when wearing N95 as an estimate of the lower border of the chin. These issues may have affected the results while using ImageJ to measure facial ratios, ultimately affecting the comparison to the existing literature. Despite these limitations, the estimated ratio for each subject obtained from the thermal images was consistent with our record of the subject’s face shape during the study, providing a basis for analysis. The majority of the subjects have round and short faces with wide cheekbones.

### Face temperature

**Fig 4:**
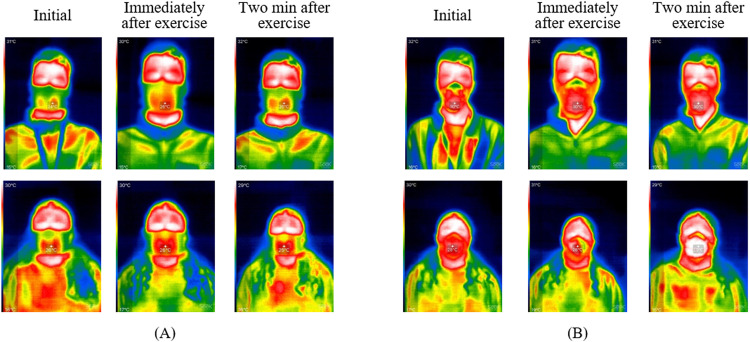
Example thermal images when wearing (A) Hero and (B) N95. Top rows in (A) and (B) are the images for Subject #6, and the bottom rows are the images for Subject #17.

**Fig 5:**
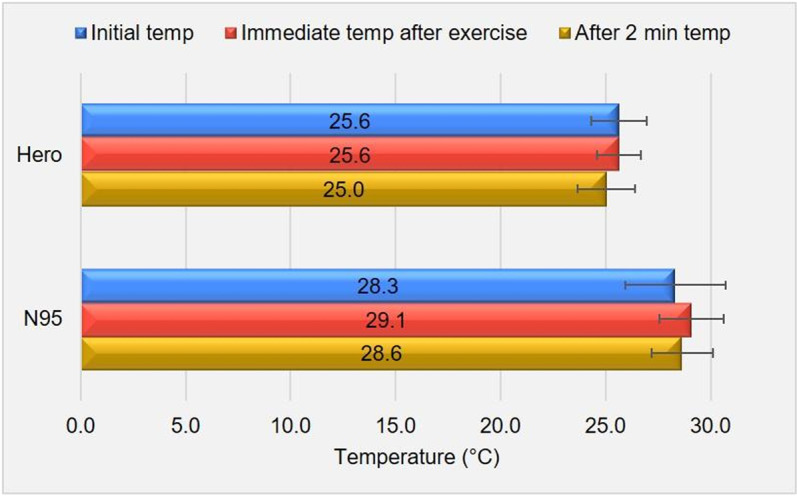
The mean temperature at each time point while wearing Hero and N95.

Example thermal images can be seen in Fig 4, where images obtained at the three time points while wearing Hero (Fig 4A) and N95 (Fig 4B) for subjects #6 and #17 are shown. Fig 5 shows the mean temperature of all subjects at each time point for Hero and N95. The temperatures obtained before exercise while wearing an N95 mask were consistent with those reported in the previous study by Scarano et al. [24]. The ANOVA test for the Hero temperatures at three different time points reveals a *p*-value of 0.20, indicating that the exercise did not result in significant temperature changes when wearing Hero. The ANOVA test for the N95 temperature reveals a *p*-value of 0.38, indicating that the exercise did not result in significant temperature changes either when wearing N95.

Comparing face temperatures wearing N95 and Hero, we obtained *p*-values of 1.3×10^-4^, 8.5×10^-9^, and 1.2×10^-8^ for the time points of the initial (before the exercise), immediately after the exercise, and two minutes after the exercise, respectively. With a significance level of 0.05, we can conclude that there is a significant difference between the mean temperatures obtained at the same time points for Hero and N95. The *p*-value obtained by comparing the mean temperatures recorded at all time points when wearing Hero with those recorded when wearing the N95 mask is 1.7×10^-18^. With a significance level of 0.05, we can conclude that there is a significant difference between the mean temperatures for Hero and N95. At all of the time points, the face temperatures are significantly lower when wearing Hero. While studies on indoor thermal comfort indicated variability in the comfort temperature based on thermal sensation votes, ranging from 21.8 to 26.9 °C [20,25], and factors such as mask wearing [20] could affect the sensation, the overall lower face temperature observed at the mask-wearing region when wearing Hero suggests better alignment with the reported comfort range, providing a quantifiable basis for improved wearability.

**Fig 6:**
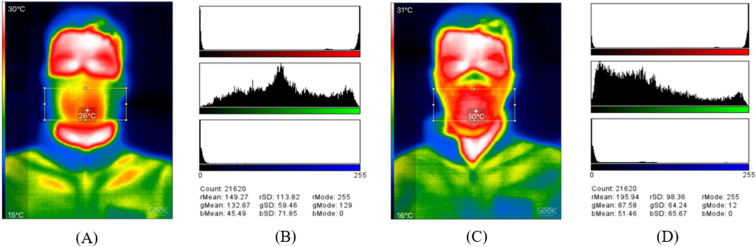
Thermal image analysis for heat intensity and distribution. (A) Selected (squared) ImageJ region of interest (ROI) of the mask-wearing region on the thermal image of Subject #6 immediately after the exercise when wearing Hero, (B) Color histograms of the ROI in Fig A, (C) Selected ROI of the mask-wearing region on the thermal image of Subject #6 immediately after the exercise when wearing N95, and (D) Color histograms of the ROI in Fig C. The horizontal axis of the histograms represents the intensity, while the vertical axis represents the number of pixels. On the panel below the histograms, the “Mean” shows average intensity values of the “r” (red), “g” (green), and “b” (blue) colors in the ROI. The “SD” is the standard deviation of the intensity of each color. The largest “Mode” value indicates the most frequently occurring color in the ROI.

To enable easy analysis of heat intensity and distribution, the thermal image of Subject #6 immediately after the exercise wearing the Hero was further analyzed and is shown in Fig 6A as an example. A region of interest (ROI) was selected to represent the mask-wearing region that was likely to be affected by the temperature changes due to the exercise. The red color channel in a color histogram (Fig 6B) provides a useful metric for assessing heat, as the higher temperature in this region is represented as red in the thermal image. For comparison, the thermal image and color histogram for Subject #6 immediately after exercise wearing the N95 is shown in Figs 6C and 6D, respectively. The panels in Figs 6B and 6D provide the mean intensity values of red, green, and blue in the ROI. The standard deviation (SD) shows the spread of the degree of variation in the pixel densities (intensities) for each color. The mode shows the most frequently occurring intensity value of each color, therefore indicating the most frequently occurring color in the ROI.

For both Hero and N95, the quantity of red pixels in the mask-wearing region after the exercise increased and concentrated, compared to that before the exercise, as shown in Fig 4. When comparing the ROIs after the exercise, there is a higher heat and heat concentration when wearing N95, as observed from the thermal images and indicated by the higher mean intensity value and smaller SD value of the red channel in Fig 6D than in Fig 6B. Similar trends can be seen in S2 Fig, where the thermal images of Subject #17 immediately after the exercise are shown. The results from the thermal image analysis are congruent with the qualitative results from the post-test questionnaire. The majority of the subjects felt that it was cooler and drier when wearing Hero than N95. This allows Hero to be more comfortable to wear and increases its functionality by improving the wearing experience.

As we noticed that the face shape affected the fit and comfort of using the current Hero design. To further study the interactions between Hero/N95 and subjects and decide whether the subjects’ perception of warmth was affected by this factor, an analysis was performed on the temperature in relation to the subject face shape which is indicated by the facial ratio. Table 2 shows the average temperature of masks at different time points for the different facial ratio ranges (<1.4, 1.4~1.499, and >1.499). The ANOVA test indicates that facial ratio did not significantly impact (*p*>0.05) temperature wearing either Hero or N95, when comparing the temperatures obtained at the same time point wearing the same type of mask. Rather, it is still the type of mask (Hero or N95) that significantly impacts the temperature.

**Table 2:** Temperature comparison based on different facial ratios.

	Altus Hero 1			N95		
**Initial temp (°C)**	**Immediate temp after exercise (°C)**	**After 2 min temp (°C)**	**Initial temp (°C)**	**Immediate temp after exercise (°C)**	**After 2 min temp (°C)**
**<1.4**	25.7 ± 1.25	25.7 ± 1.60	25.9 ± 1.68	28.3 ± 2.14	29.6 ± 1.27	28.9 ± 1.21
**1.4~1.499**	26.2 ± 1.47	25.8 ± 0.41	24.8 ± 0.75	27.8 ± 3.66	29.5 ± 1.76	28.7 ± 1.63
**>1.499**	25.2 ± 1.30	25.4 ± 0.88	25.6 ± 1.24	28.7 ± 1.73	28.4 ± 1.51	28.4 ± 1.67

In addition to the facial ratio, gender was a potential factor for varying the face temperatures and user experience as implied by the significantly different scores for “less heat retention” given by different genders, shown in Table 1. Table 3 shows the mean face temperatures for different genders. However, the mean temperatures for female and male subjects were not significantly different (*p*>0.05), suggesting that gender does not play a substantial role in determining the actual temperature. The significantly different scores for “less heat retention” might be attributed to perception differences between genders [26].

**Table 3:** Temperature comparison based on gender.

	Altus Hero 1			N95		
**Initial temp (°C)**	**Immediate temp after exercise (°C)**	**After 2 min temp (°C)**	**Initial temp (°C)**	**Immediate temp after exercise (°C)**	**After 2 min temp (°C)**
**Female**	25.7 ± 1.14	25.4 ± 1.16	24.9 ± 1.29	28.4 ± 1.69	28.8 ± 0.76	28.8 ± 1.51
**Male**	25.5 ± 2.71	26.0 ± 1.67	25.4 ± 1.31	28.1 ± 1.89	29.6 ± 1.19	28.4 ± 1.77

### Blood oxygen saturation

**Fig 7:**
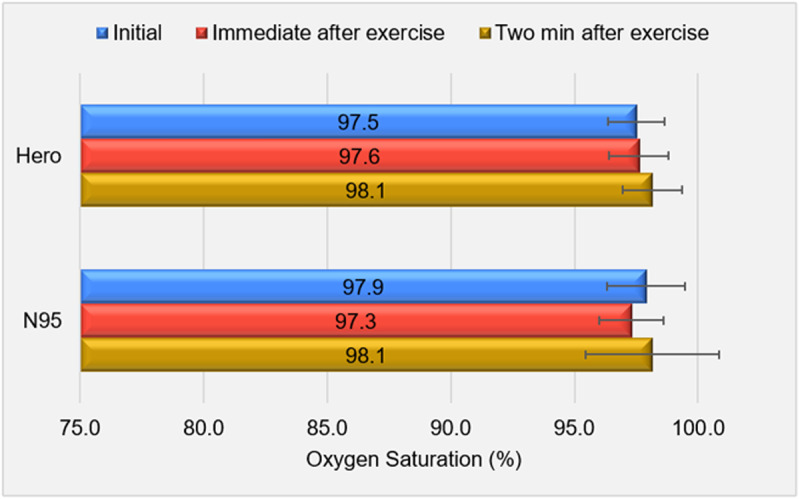
The mean blood oxygen levels (oxygen saturation) during the use of Hero and N95 at different time points.

Fig 7 shows blood oxygen saturation data for Hero and N95. An ANOVA test indicated that there is no significant difference (*p*>0.05) among the mean values of blood oxygen levels at different time points while wearing either Hero or N95, aligning with findings from previous studies with surgical masks and N95 [27,28]. Using a two-tailed paired *t*-*t*est for a comparison between the Hero and N95 blood oxygen levels, we obtained *p*-values of 0.036, 0.18, and 1.00 for the time points of the initial, immediately after the exercise, and two minutes after the exercise, respectively. There is a significant difference between the means of blood oxygen levels obtained before exercise, but no significant difference at the other time points. It was noticed that there might be inaccurate readings due to some subjects wearing nail polish. The *p*-value obtained by comparing all blood oxygen levels for Hero to that for N95 is 0.87, indicating that there is no significant difference.

## Discussion

The discomfort of wearing an N95 is primarily caused by the accumulation of hot and humid air near the face due to both reduced effective air exchange rate and poor heat transfer [29]. Hero utilizes UVC to inactivate pathogens rather than filtering based on particle sizes, which allows free airflow to enter and exit the mask-wearing region. Our results indicated that this design greatly contributes to reducing heat and moisture retention for improved comfort of wearing. Future studies on the relationship between perceived comfort, environmental factors (e.g., room ventilation and humidity), and humidity and temperature measurement with larger sample sizes and prolonged mask wear in real-world settings (e.g., healthcare environments) will provide further insights into user needs and mask performance.

The blood oxygen levels collected show little variation (<1%) between Hero and N95, indicating that neither Hero nor N95 would impair breathing. It is noted that the current study included only adult subjects without any respiratory diseases. With free airflow, the design of Hero might benefit those with breathing difficulties or children particularly during physical activities, which is a current issue reported for N95 [30]. We plan to recruit a larger group of subjects with more varieties in the next phase of the test to study these effects.

We collected user preferences and feedback which sheds light on future design improvements for novel face masks to enhance usability and compliance. The first major improvement may come from the design to fit different facial ratios. Hero fits best on longer and narrower faces when the edges of the mask can sit above the cheekbone, fit along the cheek, and rest on the bottom of the chin. The subject population demonstrated a higher discomfort for rounder and shorter face shapes with wide cheekbones. For these subjects, the top of the mask crossed over the cheekbone and sat immediately under it. This caused excessive pressure on the nose and cheeks and left red markings on the face. In addition, some subjects claimed the mask was “too bulky” and blocked their lower peripheral vision by approximately 25%. This was noted to be more common in users with shorter faces. Future studies should incorporate detailed anthropometric measurements in larger-scale studies to better understand the relationship between facial structures and mask design. This will aid in designing various models or a model with more flexibility that can fit different users better.

Considering the needs in healthcare settings, compatibility with eyeglasses and eye protection equipment needs to be considered. Though less eyeglasses fogging was reported for Hero than N95, the bulkiness of Hero and face placement impaired the ability of eyeglasses users to properly fit their glasses above the mask. Efforts to slim down the front panel while not compromising the free-breathing experience or make additional or flexible models may further improve the overall comfort and functionality.

Hero’s overall shape and material had little negative impact on comfort. However, it received some complaints in the don/doff which took longer than expected and required a mirror to accomplish. This could be increasingly difficult for users with tremors, difficulties with wrist mobility, or overall reduced dexterity. Additionally, at least two subjects needed different strap and mask sizes. Due to the wiring located along the straps connecting the battery compartment located at the back to the front panel, these straps could not be removed and switched out for the appropriate size. This could be amended by having a removable attachment and port for connecting the battery compartment. The location of the battery compartment may need to be adjusted to enable easier donning and doffing. As battery technology advances, incorporating batteries with higher energy densities, reduced weight, and smaller sizes will enhance the comfort of wearing [31].

Other comments by subjects focused on the ability to communicate while wearing Hero. The subjects claimed they felt their voice was muffled or blocked. This could be attributed to the material that the front panel was constructed out of and the good seal of the mask. Some subjects claimed they didn’t like the transparency and would prefer an opaque mask. This could be resolved by offering different model types and customization options.

Environmental factors were suggested by the subjects, as some Hero components are made out of plastics and synthetic materials. As a reusable mask, the environmental impacts can be mitigated through Hero’s reusability. Research and considerations into more environmentally conscious materials would improve user satisfaction and the impact of the device after its use.

## Conclusion

Overall, this study showed an improved user experience with the novel design of Hero, compared to N95. We identified several design considerations that help improve the user experience and compliance, including the fit to different face shapes and ratios, features to enable better communication and breathability, comfort of wearing, compatibility to eyeglasses or goggles, ease of don/doff, environmental factors, and wearer customization. These design considerations will provide manufacturers with the necessary information for future face mask designs with enhanced user experience. Further studies will include larger sample sizes and longer-term usability studies for summative studies and evaluate the clinical implications of these usability factors in real-world healthcare settings.

The global market for reusable face masks is expected to grow at a compound annual growth rate of 26.8%, reaching $11.54 billion by 2028 [32]. This growth is driven by trends such as transparent and communication-friendly masks, modular and interchangeable components, UVC sterilization, and integrated filters and air purification systems. Despite these advancements, few studies have examined the user experience of masks incorporating these novel technologies or developed methodologies to evaluate their usability. This study addresses these gaps by providing critical and universal design considerations for future face mask development and introducing innovative evaluation methodologies to assess user experience, which may contribute to enhanced compliance and overall mask effectiveness.

## Supporting information

S1 Appendix Tests on the functionality and safety of Hero. (DOCX)

S2 Appendix Modified NIOSH N95 respirator quantitative fit test protocol. (DOCX)

S3 Appendix Post-test questionnaire. (PDF)

S1 Table Mask sizes, fit factors, and face dimensions. (DOCX)

S1 Fig Distribution of subject age demographic. (DOCX)

S2 Fig Thermal image analysis for heat intensity and distribution. (DOCX)
