# Supplementary material for: A comparison of fit, heat stress, oxygen saturation and comfort between a novel reusable mask and disposable N95 respirator
Source: PLoS One. 2025 Apr 16;20(4):e0321538. doi: 10.1371/journal.pone.0321538 (PMC12002532; doi:10.1371/journal.pone.0321538)
Supplement: S1 Appendix — (DOCX) [file pone.0321538.s001.docx]

**Supporting Information**

**Tests on the efficacy and safety of Hero**

(a) Inactivation against viral and bacterial pathogen

Nelson Labs (2020). Study Number 1307847-S01, Bacterial Filtration Efficiency (BFE) at an Increased Challenge Level GLP Report, Nelson Labs, August 13, 2020

Nelson Labs (2021). Study Number 1460600-S01, Viral Filtration Efficiency (BFE) at an Increased Challenge Level GLP Report, Nelson Labs, November 6, 2021

Nelson Labs (2022a). Study Number 1496807-S01, Viral Filtration Efficiency (BFE) at an Increased Challenge Level GLP Report, Nelson Labs, March 18, 2022

Nelson Labs (2022b). Study Number 1496806-S01, Bacterial Filtration Efficiency (BFE) at an Increased Challenge Level GLP Report, Nelson Labs, March 21, 2022

(b) Ultraviolet-C (UVC) safety

Intertek (2020). Report number 104387632CRT-001, Non-standard UVC Light Leakage Test, Intertek, December 29, 2020

Intertek (2021). Report number 104701658GRR-001. Ozone Emissions Testing of Household Electrostatic Air Cleaners on BOLB SMD6060, Intertek, July 12, 2021.
