# Supplementary material for: A comparison of fit, heat stress, oxygen saturation and comfort between a novel reusable mask and disposable N95 respirator
Source: PLoS One. 2025 Apr 16;20(4):e0321538. doi: 10.1371/journal.pone.0321538 (PMC12002532; doi:10.1371/journal.pone.0321538)
Supplement: S2 Appendix — (DOCX) [file pone.0321538.s002.docx]

**Supporting Information**

**Modified NIOSH N95 respirator quantitative fit test protocol**

TSI ambient aerosol condensation nuclei counter (CNC) quantitative fit testing protocol was adopted for use on Altus Hero 1 mask (Hero). The ambient aerosol CNC quantitative fit testing (Portacount TM) protocol quantitatively fit tests respirators with the use of a probe. The probed respirator (Altus Hero 1) is only used for quantitative fit tests. A probed respirator has a sampling device, installed on the respirator, that allows the probe to sample the air from inside the mask. A probed respirator is required for each make, style, model, and size, and can be obtained from the respirator manufacturer or distributor. The CNC instrument manufacturer, TSI Inc., also provides probe attachments (TSI sampling adapters) that permit fit testing in different respirators. A minimum fit factor pass level of at least 50 is necessary for a half-mask respirator such as Altus Hero 1 when using the Portacount model 8038. The entire testing procedure was explained to the test subjects prior to the conduct of the test. The procedure is:

(a) Preparing fit test using purpose-built Altus Hero 1 mask for quantitative fit test. Estimate the size of the mask to be used for the user. Install 3M™ 7093 Particulate Filter (P100-rated) or equivalent filter cartridges to the vent on each side of the fit test mask.

(b) Portacount Fit Test Procedure (according to Title 8 California Code of Regulations § 5144 Appendix A Part I. OSHA-Accepted Fit Test Protocols Paragraph Section C. 3.)

(1) Check the respirator (thereafter Altus Hero 1 mask in our test) to make sure the sampling probe and line are properly attached to the facepiece and that the respirator is fitted with a particulate filter capable of preventing significant penetration by the ambient particles used by the fit test (e.g. NIOSH 42 CFR 84 series 100 particulate filter). We used 3M™ 7093 Particulate Filter in our test.

(2) Instruct the person to be tested to don the respirator for five minutes before the fit test starts. This purges the ambient particles trapped inside the respirator and permits the wearer to make certain the respirator is comfortable. This individual shall already have been trained on how to wear the respirator properly.

(3) Check the following conditions for the adequacy of the respirator fit: Chin properly placed; Adequate strap tension, not overly tightened; Fit across nose bridge; Respirator of proper size to span distance from nose to chin; Tendency of the respirator to slip; Self-observation in a mirror to evaluate fit and respirator position.

(4) Have the person wearing the respirator do a user seal check. If leakage is detected, determine the cause. If leakage is from a poorly fitting facepiece, try another size of the same model respirator.

(5) Follow the manufacturer's instructions for operating the Portacount and proceed with the test.

(6) The test subject shall be instructed to perform the test exercises of normal breathing, deep breathing, head side-to-side, head up-and-down, talking, bending over, and normal breathing again.

(7) After the test exercises, the test subject shall be questioned by the test conductor regarding the comfort of the respirator upon completion of the protocol. If it has become unacceptable, another size/model of respirator shall be tried.

(c) Portacount Test Instrument.

(1) The Portacount will automatically stop and calculate the overall fit factor for the entire set of exercises. The overall fit factor is what counts. The “Pass” or “Fail” message will indicate whether or not the test was successful. If the test was a “Pass”, the fit test is over.

(2) Since the pass or fail criterion of the Portacount is user-programmable, the test operator shall ensure that the pass or fail criteria meet the requirements for minimum respirator performance.

(3) A record of the test needs to be kept on file, assuming the fit test was successful. The record contains the test subject's assigned ID #; overall fit factor; make, model, style, and size of respirator used; and date tested.
