# Supplementary material for: A comparison of fit, heat stress, oxygen saturation and comfort between a novel reusable mask and disposable N95 respirator
Source: PLoS One. 2025 Apr 16;20(4):e0321538. doi: 10.1371/journal.pone.0321538 (PMC12002532; doi:10.1371/journal.pone.0321538)
Supplement: S3 Appendix — (PDF) [file pone.0321538.s003.pdf]

Post Test Questionnaire

Untitled Section

1. Participant ID assigned to you

____

2. Age

____

3. Gender

Mark only one oval.

- ( ) Female
- ( ) Male
- ( ) Prefer not to say

About the study

4. Please confirm that you are voluntarily participating in the user study of a product prototype not yet approved by the FDA?

Mark only one oval.

- Yes
- No

5. Do you suffer from claustrophobia or anxiety when wearing a respirator?

Mark only one oval.

- Yes
- No

6. Have you watched the training video for wearing the Altus Hero 1 mask?

Mark only one oval.

- Yes
- No

7. Do you believe that you have received adequate training to wear the Hero 1 mask in addition to the video?

Mark only one oval.

- Yes
- No

8. Do you believe that you are in a calm mood to provide objective evaluation of the masks?

Mark only one oval.

- Yes
- No

Fit Test

9. Do you believe that you have received adequate instructions for the self-check fit test procedure for Hero 1 mask?

Mark only one oval.

- Yes
- No

Safety-related Questions

10. Do you understand the principle of disinfection for Hero 1 and its advantages against N95 type respirators?

Mark only one oval.

- Yes
- No

11. Do you believe you received adequate instructions regarding the various warning functions of the mask?

Mark only one oval.

- Yes
- No

12. Would you be able to tell when you need to exchange for a fresh battery?

Mark only one oval.

- Yes
- No

13. Were you able to hear the voice warnings clearly?

Mark only one oval.

- Yes
- No

14. Were you able to feel the haptic feedback (vibration)?

Mark only one oval.

- Yes
- No

15. Do you understand the risk of Ultraviolet-C (UV-C) leakage and how to prevent it?

Mark only one oval.

- Yes
- No

16. Do you understand the risk of the heatsink surface exceeding 41 °C (106 F) and how to avoid improper handling?

Mark only one oval.

- Yes
- No

17. Overall, have your safety concerns with this mask been adequately addressed?

Mark only one oval.

- Yes
- No

18. Do you have other safety concerns that you think need to be addressed?

Mark only one oval.

- Yes
- No

19. If your answer is YES for the above question, please list the safety concerns that you think need to be addressed.

____
____
____
____
____

Hero 1 vs N95 comparison

When comparing N95 to Hero 1 masks, please indicate the one which is more comfortable by these following measures: 1=strongly prefer Hero 1, 3=neutral, 5=strongly prefer N95

20. Which mask allows better communication?

Mark only one oval.

	1	2	3	4	5	
Strongly prefer Hero 1 mask	◯	◯	◯	◯	◯	Strongly prefer N95

21. Which mask offers superior breathability (lower air resistance) when walking or climbing up stairs?

Mark only one oval.

	1	2	3	4	5	
Strongly prefer Hero 1 mask	◯	◯	◯	◯	◯	Strongly prefer N95

22. Which mask offers less heat retention and you believe will be more comfortable to wear in hot weather?

Mark only one oval.

	1	2	3	4	5	
Strongly prefer Hero 1 mask						Strongly prefer N95

23. Which mask fogs the eye glasses less? (if you are a eye glasses wearer, please answer this question; If you are NOT, please skip this question)

Mark only one oval.

	1	2	3	4	5	
Strongly prefer Hero 1 mask						Strongly prefer N95

24. Which mask allows you to feel less moisture accumulation?

Mark only one oval.

	1	2	3	4	5	
Strongly prefer Hero 1 mask						Strongly prefer N95

25. You believe which mask is preferred for long duration wear, e.g. international flights?

Mark only one oval.

	1	2	3	4	5	
Strongly prefer Hero 1 mask	◯	◯	◯	◯	◯	Strongly prefer N95

26. Please provide top 3 improvement suggestions for Hero 1 mask

This content is neither created nor endorsed by Google.

Google Forms
