# Supplementary material for: A comparison of fit, heat stress, oxygen saturation and comfort between a novel reusable mask and disposable N95 respirator
Source: PLoS One. 2025 Apr 16;20(4):e0321538. doi: 10.1371/journal.pone.0321538 (PMC12002532; doi:10.1371/journal.pone.0321538)
Supplement: S1 Table — (DOCX) [file pone.0321538.s004.docx]

**Supporting Information**

**S1 Table.** **Mask size used by each subject, fit factor obtained during quantitative fit test, and ImageJ-based measurement of face height and width.**

| **Subject #** | **Mask size** | **Fit factor^a^** | **Vertical measurement (pixels)** | **Horizontal measurement (pixels)** | **Ratio^b^ (vertical/horizontal)** |
| --- | --- | --- | --- | --- | --- |
| 1 | M | 164 | 314.10 | 202.04 | 1.55 |
| 2 | S | 200+ | 229.02 | 153.01 | 1.50 |
| 3 | M | 85 | 247.00 | 161.01 | 1.53 |
| 4 | L | 91 | 289.00 | 176.05 | 1.64 |
| 5 | L w/ M straps^c^ | 93 | 236.10 | 149.05 | 1.58 |
| 6 | M | 51 | 364.01 | 187.00 | 1.95 |
| 7 | S | 200+ | 232.02 | 179.14 | 1.30 |
| 8 | M | 200+ | 250.10 | 160.08 | 1.56 |
| 9 | L | 65 | 226.00 | 157.00 | 1.44 |
| 10 | M | 151 | 287.09 | 206.00 | 1.39 |
| 11 | M | 74 | 251.10 | 196.00 | 1.28 |
| 12 | M | 157 | 257.05 | 170.03 | 1.51 |
| 13 | M | 63 | 262.01 | 173.00 | 1.51 |
| 14 | M w/ L straps^c^ | 63 | 305.03 | 212.02 | 1.44 |
| 15 | M | 148 | 218.01 | 154.00 | 1.42 |
| 16 | M | 174 | 293.00 | 207.01 | 1.42 |
| 17 | M | 116 | 247.02 | 181.00 | 1.36 |
| 18 | S | 39 | 285.06 | 187.00 | 1.52 |
| 19 | M | 173 | 310.03 | 261.01 | 1.19 |
| 20 | M | 50 | 291.03 | 200.00 | 1.46 |
| 21 | S | 200+ | 269.05 | 198.00 | 1.36 |
| 22 | M | 200+ | 239.01 | 174.07 | 1.37 |

^a^ An overall fit factor (the mean of fit factors obtained from all test exercises performed by a subject during the quantitative fit test) of 0-200+ was given for each subject while wearing Hero. The overall fit factors over 200 were displayed as 200+ on the TSI Portacount Fit Tester. A number equal to or larger than 50 was defined as a “PASS” criterion for the fit test.

^b^ Since the measurement was based on the thermal images, the pixel number does not represent the actual dimension of the faces, and the measurement was affected by the quality of the thermal images. The total facial height was obtained between the hairline (the top border of the red region at the forehead) and the bottom border of the red mask-wearing region as an estimate of the lower border of the chin when wearing N95.

^c^ Subjects (#5 and #14) who were better fitted into a mask (e.g. sized L for #5) with adjustable straps paired with a different-sized mask (e.g. L-sized mask with straps originally paired with M-sized masks for Subject #5).
