# Supplementary figures and images for: A comparison of fit, heat stress, oxygen saturation and comfort between a novel reusable mask and disposable N95 respirator
Source: PLoS One. 2025 Apr 16;20(4):e0321538. doi: 10.1371/journal.pone.0321538 (PMC12002532; doi:10.1371/journal.pone.0321538)

**Supporting Information**

| 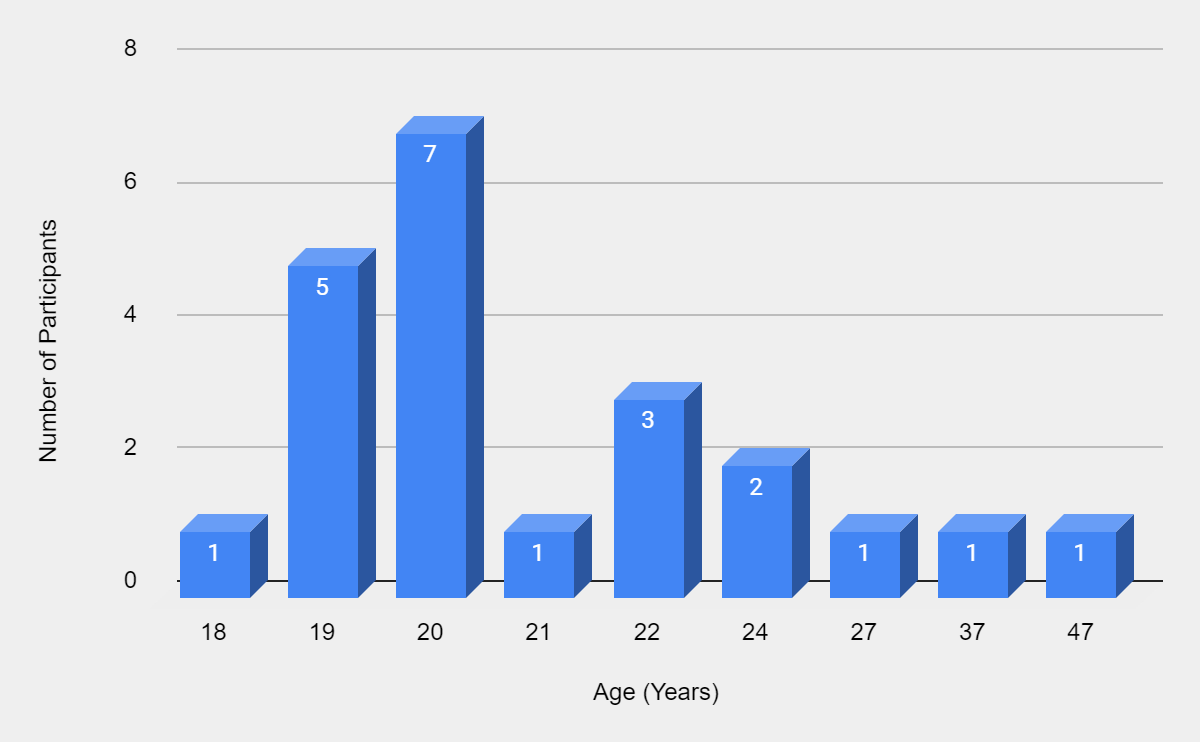 |
| --- |
| **S1 Fig. Distribution of subject age demographic.** |

Supplement: S1 Fig — (DOCX) [file pone.0321538.s005.docx]
