# Supplementary material for: A comparison of fit, heat stress, oxygen saturation and comfort between a novel reusable mask and disposable N95 respirator
Source: PLoS One. 2025 Apr 16;20(4):e0321538. doi: 10.1371/journal.pone.0321538 (PMC12002532; doi:10.1371/journal.pone.0321538)
Supplement: S2 Fig — (DOCX) [file pone.0321538.s006.docx]

**Supporting Information**

| **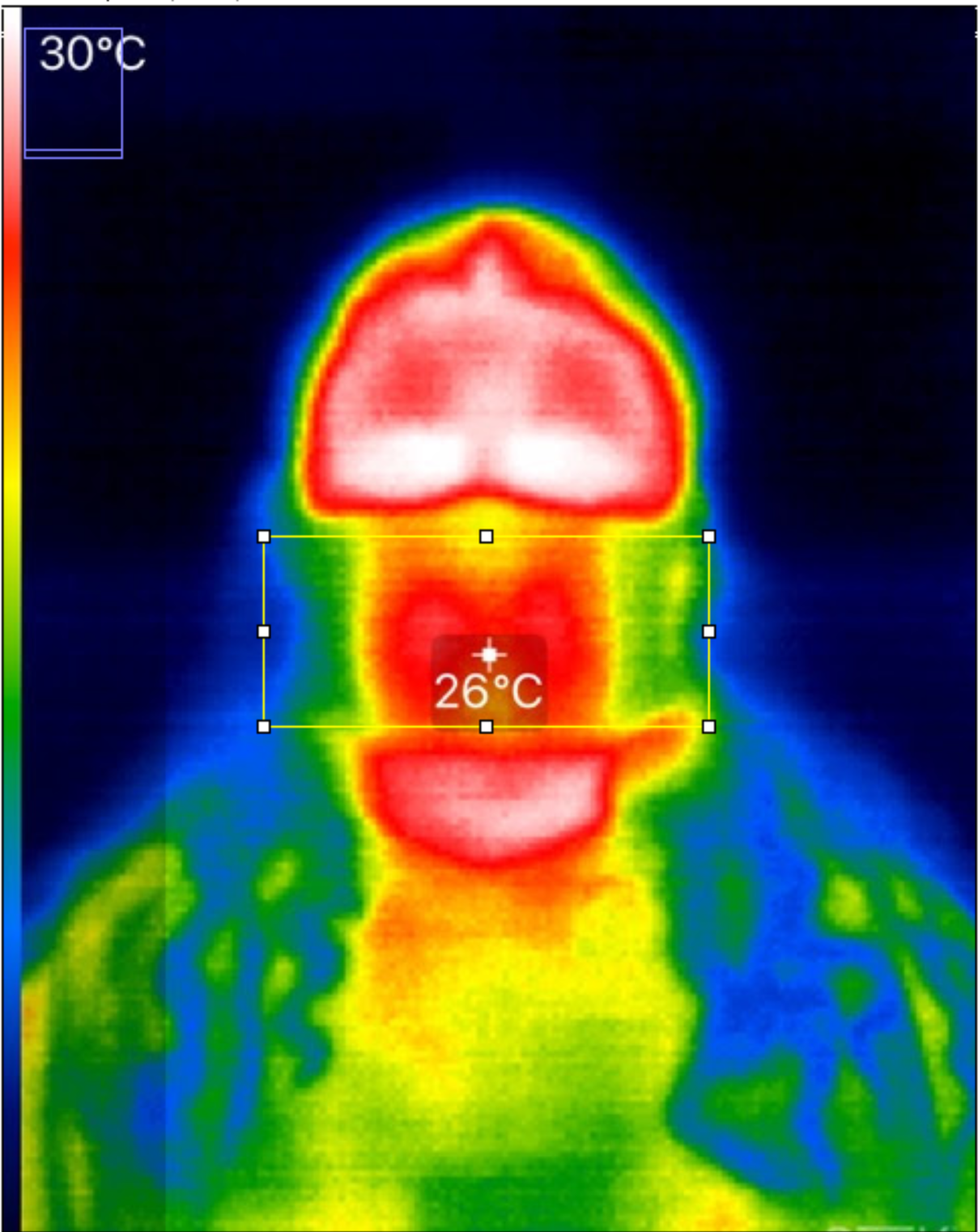** | **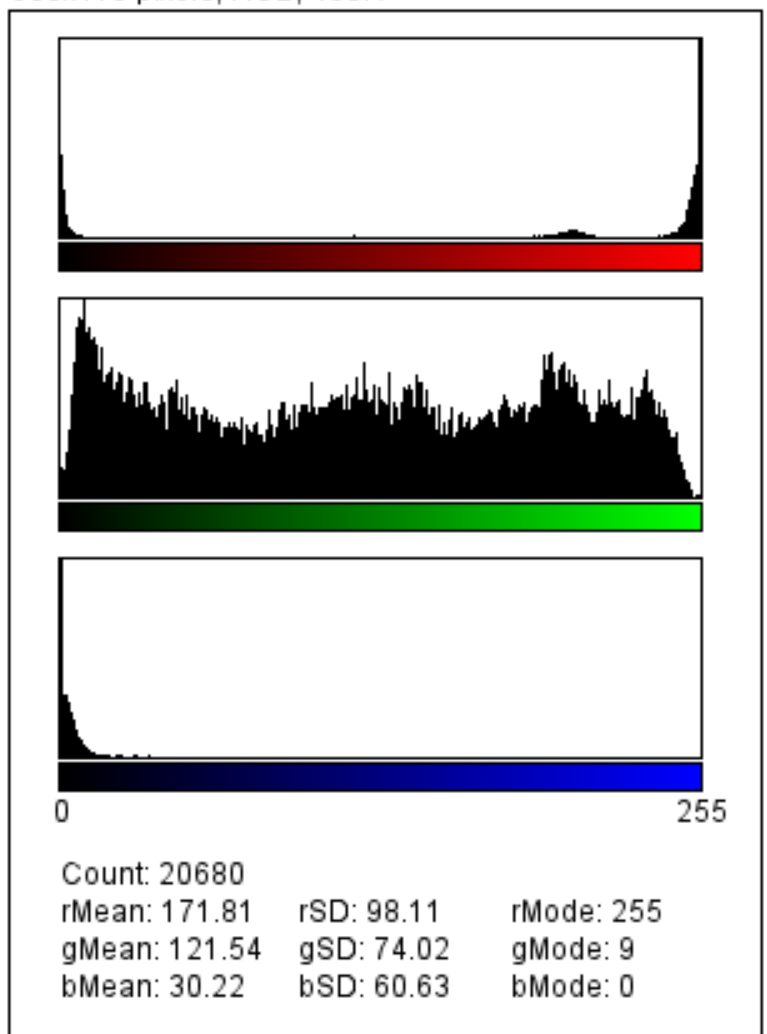** | 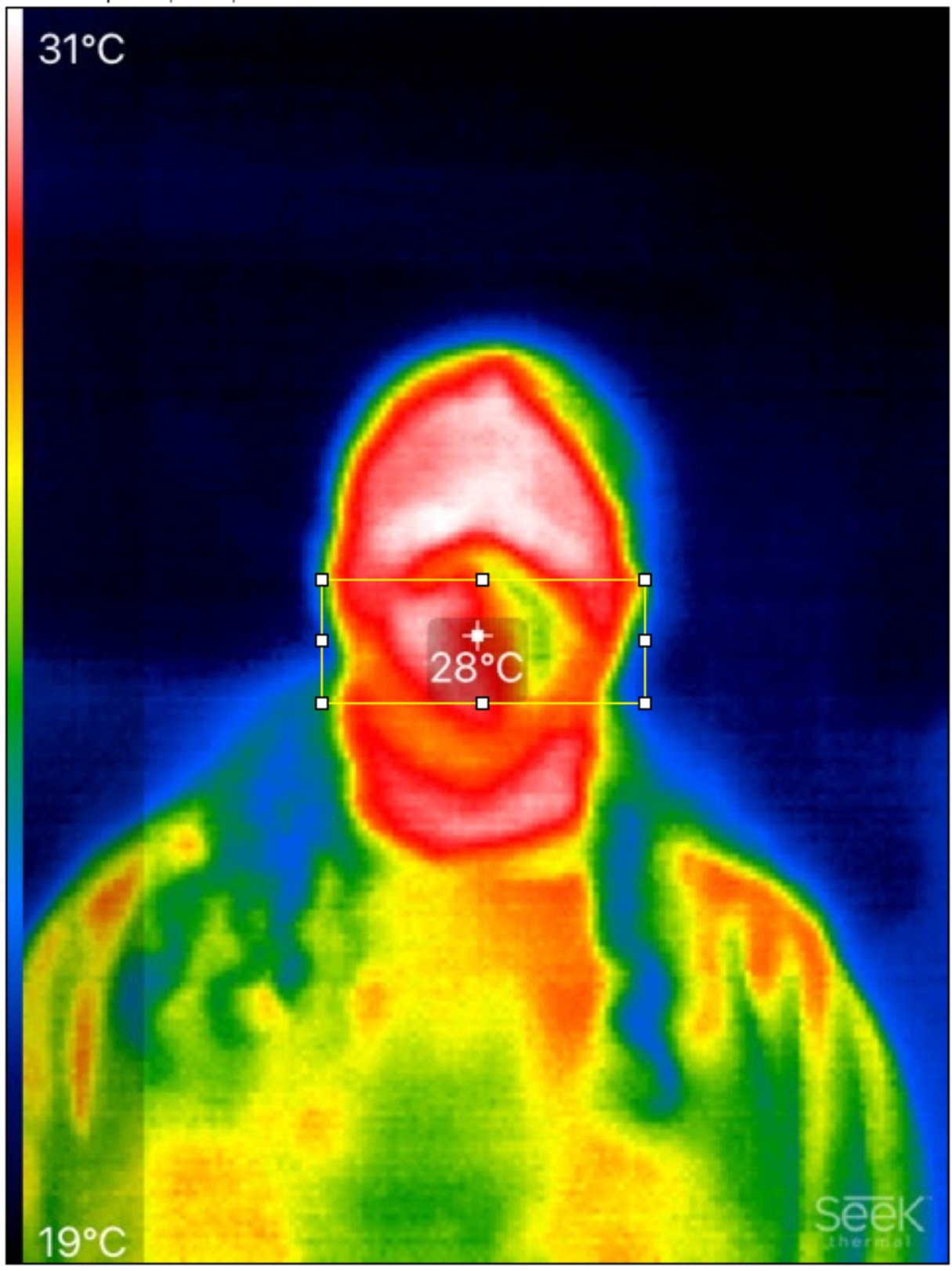 | 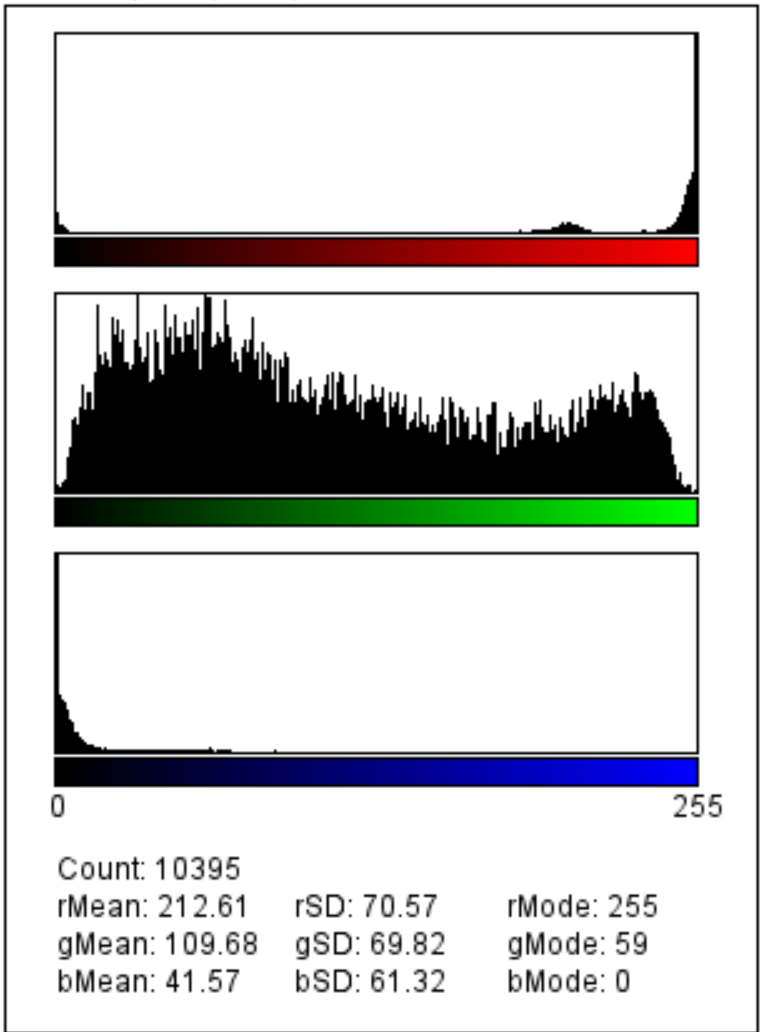 |
| --- | --- | --- | --- |
| (A) | (B) | (C) | (D) |
| **S2 Fig. Thermal image analysis for heat intensity and distribution.** (A) Selected (squared) ImageJ region of interest (ROI) of the mask-wearing region on the thermal image of Subject #17 immediately after the exercise when wearing Hero, (B) Color histograms of the ROI in Figure A. (C) Selected ROI of the mask-wearing region on the thermal image of Subject #17 immediately after the exercise when wearing N95, and (D) Color histograms of the ROI in Figure C. The horizontal axis of the histograms represents the intensity, while the vertical axis represents the number of pixels. On the panel below the histograms, the “Mean” shows average intensity values of the “r” (red), “g” (green), and “b” (blue) colors in the ROI. The “SD” is the standard deviation of the intensity of each color. The largest “Mode” value indicates the most frequently occurring color in the ROI. | | | |
